# A mechanistic model captures the emergence and implications of non-genetic heterogeneity and reversible drug resistance in ER+ breast cancer cells

**DOI:** 10.1093/narcan/zcab027

**Published:** 2021-07-09

**Authors:** Sarthak Sahoo, Ashutosh Mishra, Harsimran Kaur, Kishore Hari, Srinath Muralidharan, Susmita Mandal, Mohit Kumar Jolly

**Affiliations:** Centre for BioSystems Science and Engineering, Indian Institute of Science, Bangalore 560012, India; Undergraduate Programme, Indian Institute of Science, Bangalore 560012, India; Centre for BioSystems Science and Engineering, Indian Institute of Science, Bangalore 560012, India; Undergraduate Programme, Indian Institute of Science, Bangalore 560012, India; Centre for BioSystems Science and Engineering, Indian Institute of Science, Bangalore 560012, India; Centre for BioSystems Science and Engineering, Indian Institute of Science, Bangalore 560012, India; Department of Biotechnology, Indian Institute of Technology Madras, Chennai 600036, India; Centre for BioSystems Science and Engineering, Indian Institute of Science, Bangalore 560012, India; Centre for BioSystems Science and Engineering, Indian Institute of Science, Bangalore 560012, India

## Abstract

Resistance to anti-estrogen therapy is an unsolved clinical challenge in successfully treating ER+ breast cancer patients. Recent studies have demonstrated the role of non-genetic (i.e. phenotypic) adaptations in tolerating drug treatments; however, the mechanisms and dynamics of such non-genetic adaptation remain elusive. Here, we investigate coupled dynamics of epithelial–mesenchymal transition (EMT) in breast cancer cells and emergence of reversible drug resistance. Our mechanism-based model for underlying regulatory network reveals that these two axes can drive one another, thus enabling non-genetic heterogeneity in a cell population by allowing for six co-existing phenotypes: epithelial-sensitive, mesenchymal-resistant, hybrid E/M-sensitive, hybrid E/M-resistant, mesenchymal-sensitive and epithelial-resistant, with the first two ones being most dominant. Next, in a population dynamics framework, we exemplify the implications of phenotypic plasticity (both drug-induced and intrinsic stochastic switching) and/or non-genetic heterogeneity in promoting population survival in a mixture of sensitive and resistant cells, even in the absence of any cell–cell cooperation. Finally, we propose the potential therapeutic use of mesenchymal–epithelial transition inducers besides canonical anti-estrogen therapy to limit the emergence of reversible drug resistance. Our results offer mechanistic insights into empirical observations on EMT and drug resistance and illustrate how such dynamical insights can be exploited for better therapeutic designs.

## INTRODUCTION

Emergence of drug resistance remains the biggest hurdle in clinical management of cancer. It has been largely tacitly assumed that the acquisition of genomic mutations is a necessary and sufficient condition for drug resistance. However, recent studies across multiple cancers have suggested a set of alternative non-genetic mechanisms that can facilitate the survival of cancer cells in the presence of cytotoxic therapies (1). These non-genetic mechanisms do not entail changes in genotype (underlying DNA sequence) but in the manifestation of phenotype (2,3) through epigenetic or transcriptional reprogramming and/or cell-state transitions (4–8). Unlike genetic changes which are ‘hard-wired’ and irreversibly passed to further generations, the non-genetic changes are reversible and stochastic in nature and thus not necessarily heritable. None of the existing therapies has been yet shown to be capable of outsmarting this adaptive ability of cancer cells to alter their phenotype without modifying their genotype. Instead, drug treatment can promote such cell-state transitions, thus potentially worsening the disease progression (9). Thus, despite major advancements in targeted therapy, mechanisms of non-genetic heterogeneity and reversible drug resistance remain largely elusive.

Tamoxifen was the first targeted therapy for breast cancer which was given to ER+ (estrogen receptor-positive) breast cancer patients to bind to estrogen receptor (ER) and antagonize the proliferative ability potentiated by binding of ER to growth hormone estrogen (10). Estrogen receptor alpha (ERα) is one of the two forms of ER that lies upstream to various genomic and nongenomic signaling pathways that control cellular proliferation and survival, essentially regulating the growth of normal breast tissue and tumor (10). ERα is considered as a key prognostic marker; increased response to anti-estrogen therapies (such as tamoxifen) and better patient survival are associated with higher levels of ERα (11). However, one-third of women treated with tamoxifen for 5 years have recurrent disease within 15 years, with a majority of them being metastatic (10,12). Thus, development of acquired resistance to tamoxifen limits its efficacy.

One of the molecules associated with tamoxifen insensitivity is ER-α36, a variant form of ERα (also known as ER-α66) (13,14). As compared to ER-α66, ER-α36 lacks both transcriptional activation domains (AF-1 and AF-2) but retains the DNA-binding and ligand-binding domains. ER-α36 has been associated with activating downstream signaling pathways that promote cell proliferation, highlighting its potential role in development of drug resistance against anti-estrogen treatment (15–17). Mechanistically, ER-α36 has been shown to be transcriptionally activated by tamoxifen (18) but inhibited by ER-α66 (19).

Another process associated with tamoxifen resistance is epithelial–mesenchymal transition (EMT) (20,21), a cell plasticity program involved with drug resistance across cancers (22,23). Loss of ERα induced changes concomitant with EMT (24). Consistently, tamoxifen-resistant cells were seen to grow loose colonies with weak cell–cell adhesion, typical of EMT (25). On the other hand, EMT-inducing transcription factors such as SLUG, ZEB1 and SNAIL are known to drive tamoxifen resistance (26–28). This bidirectional coupling between ERα and EMT pathways is reinforced by analysis of 118 breast tumor specimens showing specific association of ER-α36 with various EMT markers such as MMP9, SNAIL1 and VIM (29). However, given that EMT is a reversible phenomenon where cancer cells can stably acquire one or more hybrid epithelial/mesenchymal phenotypes too (30), many questions remain: (i) can EMT drive acquisition of reversible resistance to tamoxifen, i.e. can the reverse of EMT—mesenchymal–epithelial transition (MET)—restore tamoxifen sensitivity? (ii) can tamoxifen resistance drive a partial or full EMT? and (iii) do cells need to undergo a full EMT to gain tamoxifen resistance, or can epithelial and hybrid E/M cells also possibly show those traits?

Here, we develop a mechanism-based model based on a gene regulatory network composed of key known regulators of EMT and tamoxifen resistance (TamR). Dynamical simulations of this network reveals different ‘attractors’ (expression patterns) that can emerge, thus enabling the (co)-existence of various states along EMT and TamR axes: ES (epithelial–Tam sensitive), ER (epithelial–Tam resistant), HS (hybrid–Tam sensitive), HR (hybrid–Tam resistant), MS (mesenchymal–Tam sensitive) and MR (mesenchymal–Tam resistant). Further, the emergent dynamics of the coupled processes of EMT and TamR facilitates either process to be able to drive another one, thus enabling cells to switch among multiple phenotypes along these interconnected axes and driving non-genetic heterogeneity. Finally, we develop a population dynamics model to decipher the contribution of intrinsic and tamoxifen-induced phenotypic plasticity and non-genetic heterogeneity in a cell population. Our simulations suggest that the long-term maintenance of TamR cells in a population can arise from many possible scenarios: (i) non-genetic heterogeneity in sensitivity to tamoxifen in the initial cell population and (b) phenotypic plasticity enabled by EMT and/or drug treatment. Thus, the emergence of TamR can be potentially curtailed through combinatorial targeting both EMT and ERα pathways.

## MATERIALS AND METHODS

### RACIPE simulations

Random Circuit Perturbation (RACIPE) (31) generates an ensemble of kinetic models for a given gene regulatory network and simulates its dynamics for a range of biologically relevant parameters and initial conditions. The input network is composed of inhibitory and activating links between each node. The expression of each node in the network is calculated through a set of Ordinary Differential Equations (ODEs) defined as follows:

$$\begin{equation*}\frac{d{X_i}}{dt} = {g_{X_i}}{\prod \limits_j}\,{H^s}\left( {X_j},\;{X_{ji}}0,{n_{ji}},\;{\lambda_{ji}} \right) -{k_{X_i}}{X_i}\end{equation*}$$

**Figure 1.**
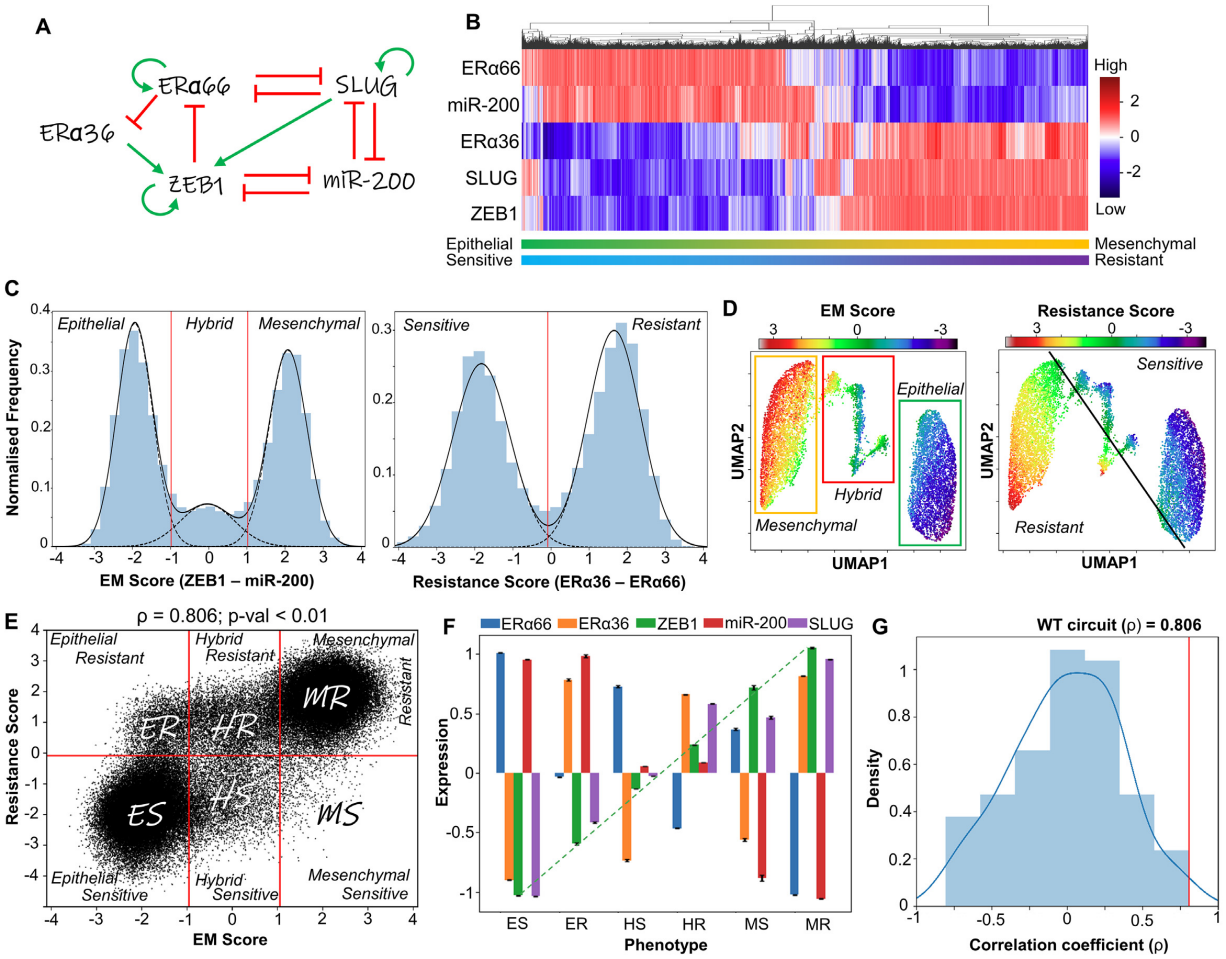
Emergent dynamics of coupled EMT-ERα signaling network. (**A**) Gene regulatory network (GRN) showing crosstalk between EMT and Estrogen receptor (ERα) signaling. Green arrows represent activation links; red hammers represent inhibition. (**B**) Heatmap of stable steady-state solutions for network shown in (A), obtained via RACIPE. (**C**) Density histogram of EM Score ( = Zeb1 – miR-200) and Resistance Score ( = ERα36 – ERα66) fitted to 3 and 2 Gaussian distributions, respectively. Red dotted lines show segregation between phenotypes: Epithelial (E), Hybrid (H) and Mesenchymal (M) for EM score, and Resistant (R) and Sensitive (S) phenotype for the latter. (**D**) UMAP dimensionality reduction plots for steady-state solutions states obtained by RACIPE colored by either EM Score or Resistance score. (**E**) Scatter plot showing corresponding EM Score and resistance score for all RACIPE solutions, and six biological phenotypes. Spearman correlation coefficient (ρ) and corresponding *P*-value are reported. (**F**) Gene expression levels in six biologically defined phenotypes. Dotted line represents the monotonic increase in levels of ZEB1 across the phenotypes. Standard deviation is plotted as error bars. (**G**) Density distribution of Spearman correlation coefficients (ρ) across an ensemble of 100 randomized versions of the GRN shown in (A). Red line shows the correlation coefficient of the wild-type network shown in (A).

Here, ${X_i},\;i \in \{ {1,2,3,4,5} \}$ is the concentration of nodes in the network, *g* is basal production rate, *k* is basal degradation rate and *H*s is shifted hill function that takes in the activatory/inhibitory links into account to determine the production rate for the node (32). The parameters corresponding to each regulatory link are λ (fold-change parameter), *n* (Hill’s coefficient) and X0 (threshold value for the Hill’s function). Network in Figure 1A was simulated for 100 000 parameter sets with default settings for parameter sampling (31). Initial condition for each node is randomly sampled from a log-uniform distribution of minimum to maximum levels of that node. RACIPE steady states obtained are z-normalized for all further analysis.

### Stochastic simulations

We simulated network in Figure 1A using Euler–Maruyama method for representative parameter sets (taken from RACIPE) corresponding to co-existence of two or three phenotypes. Equation used for simulations here are discrete forms of ODEs used by RACIPE, but with an addition of noise term $\sqrt {\Delta t} *N( {0,1} )$, where $\Delta t$ is the time step and $N( {0,1} )$ is a normal random variable with mean 0 and standard deviation 1. Using trajectories obtained via stochastic simulations of these parameter sets, we constructed and obtained a probability density (*P*) of EM-resistance score pairs and constructed a landscape by calculating the pseudo potential as $ - log( P )$ (33).

### Gene expression data analysis (clinical and single cell data)

Publicly available microarray datasets (GSE IDs mentioned appropriately) were obtained from GEO, and Spearman correlation coefficients were calculated for given genes. Single-sample gene set enrichment analysis (ssGSEA) (34) was performed on hallmark EMT, early estrogen response and late estrogen response gene signatures obtained from MSigDB (Molecular Signatures Database) (35). TamRes signature was considered to be the set of upregulated genes obtained via proteomic analysis on resistant MCF7 cells (36). EMT scoring methods—76GS, KS and MLR (37)—were used to compute EMT scores for bulk microarray datasets. Activity values computation for 10-cell and single cell datasets were done using AUCell (38). BRCA ESR1 specific regulon was obtained from GRNDb (39). Signatures for epithelial and mesenchymal phenotypes (cell lines) were taken from Tan *et al.* (40).

### Population dynamics—model formulation

In population dynamics framework, each cell has its attributes during the simulation. The three main processes during the growth/decline of a population of cells that we modelled are: proliferation, death and switching between states. We consider that the cells would be present in one of two states: sensitive (S) or resistant (R). We defined a ‘resistance score’ in the range [-6,6] that represents a cell’s fitness in the presence of an anti-estrogen drug. Cells with smaller values of ‘resistance score’ are likely to be more sensitive to the drug than cells with higher scores. We incorporated variability into the system (representative of heterogeneity) by sampling the resistance scores that are assigned to each cell from a Gaussian distribution centered a fixed value of mean but with different standard deviations. Each cell is assigned with two scores, one each for its possible sensitive (S) or resistant (R) state, former sampled from a gaussian centered at -2 and with a fixed variance and the latter sampled from a Gaussian centered at +2 and with a fixed variance. These scores are assigned during the birth of the cell and remain fixed over the course of the simulation. Each cell has an index variable that keeps track of the current status of the cell S or R. Depending on the current status of the cell, the probability of the death of cell due to the drug varies depending on the corresponding ‘sensitive’ or ‘resistant’ score.

Cell death can occur through 2 independent ways: a constant basal probability of cell death and a death due to the presence of the drug. The constant basal probability of cell death is a fixed number kept constant (at 0.1) over all simulations unless specified. The probability for drug induced cell death is dependent on the current status of the cell and the corresponding score assigned to it during its birth. To map the resistance score of each cell to a probability of death, we used a sigmoidal function. So, if a cell has a resistance score of x, then the probability of its death is given by $\frac{{{{\rm{e}}^{\rm{x}}}}}{{{{\rm{e}}^{\rm{x}}}{\rm{\;}} + {\rm{\;c}}}}$, where *c* is a constant. For our simulations, we used *c* = 0.6. Varying *c* does not change the qualitative observations of our model.

At a population level, we assume a logistic growth model for the cell population. Cells are allowed to proliferate with a probability given by:

$$\begin{equation*}{\rm Proliferation}\;{\rm rate}*\left( {1\; - \;\frac{{{\rm current}\;{\rm population}\;{\rm size}}}{{{\rm carrying}\;{\rm capacity}}}} \right)\end{equation*}$$

Proliferation rate was set at 0.91 unless specified, and carrying capacity was set at 10^5^ cells. Proliferation and basal death rates of cells do not depend on the current state of the cell. Upon cell division, daughter cells retain the status (sensitive and resistant) of the mother cell. However, to account for variability during cell division, the two scores assigned to each cell are resampled from the Gaussian distributions with specified variance (heterogeneity level).

Cells can also switch between sensitive and resistant states. Transition probability from sensitive to resistant state is given by *P*_SR_ and that from resistant to sensitive state is given by *P*_RS_. These probabilities characterize the plasticity of the system. The basal death probability, probability of proliferation, heterogeneity and transition probability constants are kept the same for a single simulation. Later, we perform a set of simulations by changing each of these parameters individually to show their effect at the population level.

### Statistical testing

We computed the Spearman correlation coefficients and used corresponding *P*-values to gauge the strength of correlations. For statistical comparison between groups, we used a two-tailed Student’s *t*-test under the assumption of unequal variances and computed significance.

Further details are given in Supplementary Data.

## RESULTS

### Crosstalk between EMT and estrogen receptor signaling result in multiple phenotypes showing broad association between EMP and drug resistance in ER+ breast cancer

First, we identified a gene regulatory network (GRN) that captures known interactions among various players involved in EMT and in tamoxifen resistance. This network incorporates the reported interactions among estrogen receptor molecules ERα66 and ERα36, and EMT players SLUG, ZEB1 and miR-200 at multiple regulatory levels (Figure 1A). ERα66 can repress ERα36 expression in an estrogen-independent manner (19) and activate its own expression (41). ERα66 can also exert controls over the EMT axis by repressing SLUG (42–44). SLUG and ZEB1 are key EMT-inducing transcription factors that can regulate the expression of ERα66 and ERα36, thereby controlling tamoxifen resistance. SLUG and ZEB1 can repress ERα66; ZEB1 can induce promoter hypermethylation, while SLUG can bind directly to the promoter as a repressor (28,45) as well as recruit LSD1 to demethylate H3K4me2 (46). On the other hand, ERα36 can enhance the expression of ZEB1 through SNAIL and/or by suppression of CDH1 (18,47,48). ZEB1 and miR-200 form a mutually inhibitory self-activatory feedback loop featuring transcriptional and translational regulatory control, and in conjunction with their interaction with SLUG, they determine the EMT phenotype of a cell (48).

To elucidate the emergent dynamics of this GRN, we simulated it using RACIPE (Random Circuit Perturbation)—a computational framework that solves a set of coupled ordinary differential equations (ODEs) to examine the various phenotypic states enabled by a GRN, by sampling an ensemble of kinetic parameter sets from a biologically relevant parameter range (31). For each distinct parameter set, it chooses initial conditions based on random sampling from within a log-uniform distribution for each node and then solves ODEs to obtain possible steady states. For some parameter sets, more than one steady state (phenotype) is achieved, suggesting possible stochastic switching among those states under the influence of noise.

Upon simulating our GRN using RACIPE, we observed multiple cell states that are visualized qualitatively as a hierarchically clustered heatmap (Figure 1B). Qualitatively, ZEB1, SLUG and ERα36 are often co-expressed and similarly, ERα66 and miR200 are co-expressed. The existence of these two major expression patterns is corroborated by *K*-means clustering (Supplementary Figure S1A). Co-expression of ERα66 and miR200 can be construed as an epithelial sensitive (ES) phenotype, given that the presence of ERα66 associates with response to an anti-estrogen drug (e.g. tamoxifen). However, in cases when ERα36 is higher, such cells are less likely to respond to an anti-estrogen compound and will exhibit drug tolerance or resistance. Thus, co-expression of ZEB1 and SLUG with ERα36 is interpreted as a mesenchymal-resistant (MR) phenotype. Further, we defined different cell states along the EMT and the drug resistance (TamR) axes. EM score is defined as the difference in normalized values of ZEB1 and miR-200; similarly, resistance score is defined as difference in normalized values of ERα36 and ERα66. Higher EM scores correspond to a mesenchymal phenotype, and higher resistance scores correspond to a resistant phenotype. A hierarchically clustered heatmap on the EM and resistance scores confirmed the existence of the ES and the MR phenotypes, along with the indication of other relatively less prevalent cell states such as epithelial-resistant (ER), hybrid-sensitive (HS) and hybrid-resistant (HR) phenotypes (Supplementary Figure S1B).

Next, we plotted a normalized frequency histogram for the EM score of steady-state solutions obtained via RACIPE. The resultant distribution was visibly trimodal in nature (Figure 1C) with two dominant peaks corresponding to epithelial and mesenchymal phenotypes. The middle peak was smaller, suggesting less abundant hybrid E/M phenotypes expressing intermediate values of ZEB1 and miR-200, as earlier postulated (48) (Supplementary Figure S1C). Similarly, the normalized frequency histogram of resistance score was bimodal, corroborated by existence of two distinct clusters along the ERα66-ERα36 axes (Supplementary Figure S1C). To better visualize the various phenotypes enabled by the GRN, we performed UMAP analysis on the steady states and colored the individual steady states by either EM score or resistance score. A qualitative comparison of the two UMAP plots revealed that the epithelial cluster was more likely to be sensitive while the mesenchymal cluster was more likely to be resistant (Figure 1D). The hybrid E/M (H) phenotype can be either resistant (R) or sensitive (S) (Figure 1D and Supplementary Figure S1D).

To further quantify the association between phenotypes on EM and resistance axes, we plotted the individual steady state solutions on these axes as a scatter plot. Discretization of the scores along these axes (see Materials and Methods section) revealed six possible distinct phenotypes (Figure 1E): ES (epithelial-sensitive), ER (epithelial-resistant), HS (hybrid-sensitive), HR (hybrid-resistant), MS (mesenchymal-sensitive) and MR (mesenchymal-resistant). The ES and MR phenotypes were the most dominant while MS was the least prevalent (Supplementary Table S1). This analysis indicates that while there exists a strong association with the EM status and drug (tamoxifen) resistance at a cellular level (ρ = 0.806, *P* < 0.01), other phenotypes—ES, HR, HS and MS—may exist too, therefore highlighting the nonbinary and semi-independent nature of EM phenotypes and its association with tamoxifen resistance.

Next, we assessed the molecular profiling of the six identified phenotypes. ZEB1 levels showed a monotonic increase in expression levels across the phenotypes ES, ER, HS, HR, MS and MR (Figure 1F). Intriguingly, SLUG levels, which was not used for classification of either EM or Resistance scores, appeared to be significantly higher in all the resistant states for a given EM phenotype, especially the hybrid E/M phenotype (Figure 1F). SLUG has previously been shown to be associated with hybrid E/M states (48). Based on these observations, SLUG can be considered as a key player in driving resistance, especially in hybrid E/M phenotypes.

Finally, we examined whether this strong association between EM and resistance scores/ phenotypes is specific to the network topology of this GRN only. To test this hypothesis, we created an ensemble of 100 randomized GRNs controlling for the total number of nodes in the network, net number of activation and inhibitory edges, and in and out degree of each node. We ran RACIPE simulations on this ensemble of networks and calculated the correlation coefficient between the EM and resistance scores. The resulting distribution is centered around 0, and the ‘wild type’ GRN (Figure 1A) had the strongest correlation among the ensemble (Figure 1G), depicting the uniqueness of this network topology to this strong association.

Further, we assessed the impact of other indirect gene regulatory links for ERα36, such as its self-activation and its ability to suppress the activity of ERα66 (16,49). Addition of these links individually or in combination resulted in qualitatively similar results in terms of clustered heatmaps, UMAP plots and EM score-resistance score scatter plots (Supplementary Figures S2 and S3A–C). Additionally, it is possible that ZEB1 can directly transcriptionally repress ERα36 along with inhibiting ERα66 (28). To examine the impact of this additional link on network dynamics, we simulated a variant network including this inhibitory link and observed that the broad association between EMT and tamoxifen resistance remains largely unchanged (Supplementary Figure S3D). Similarly, the incorporation of interactions of GATA3, a key regulator of ERα levels, with ERα66 and ZEB1 (50) also preserves the main features of our model (Supplementary Figure S3E and F; Supplementary Table S1). Together, these observations suggest that GRN in Figure 1A is sufficient to capture fundamental features of EMP and reversible drug resistance observed in ER+ breast cancer.

### Clinical data support the predicted association between EMT and loss in ERα activity

**Figure 2.**
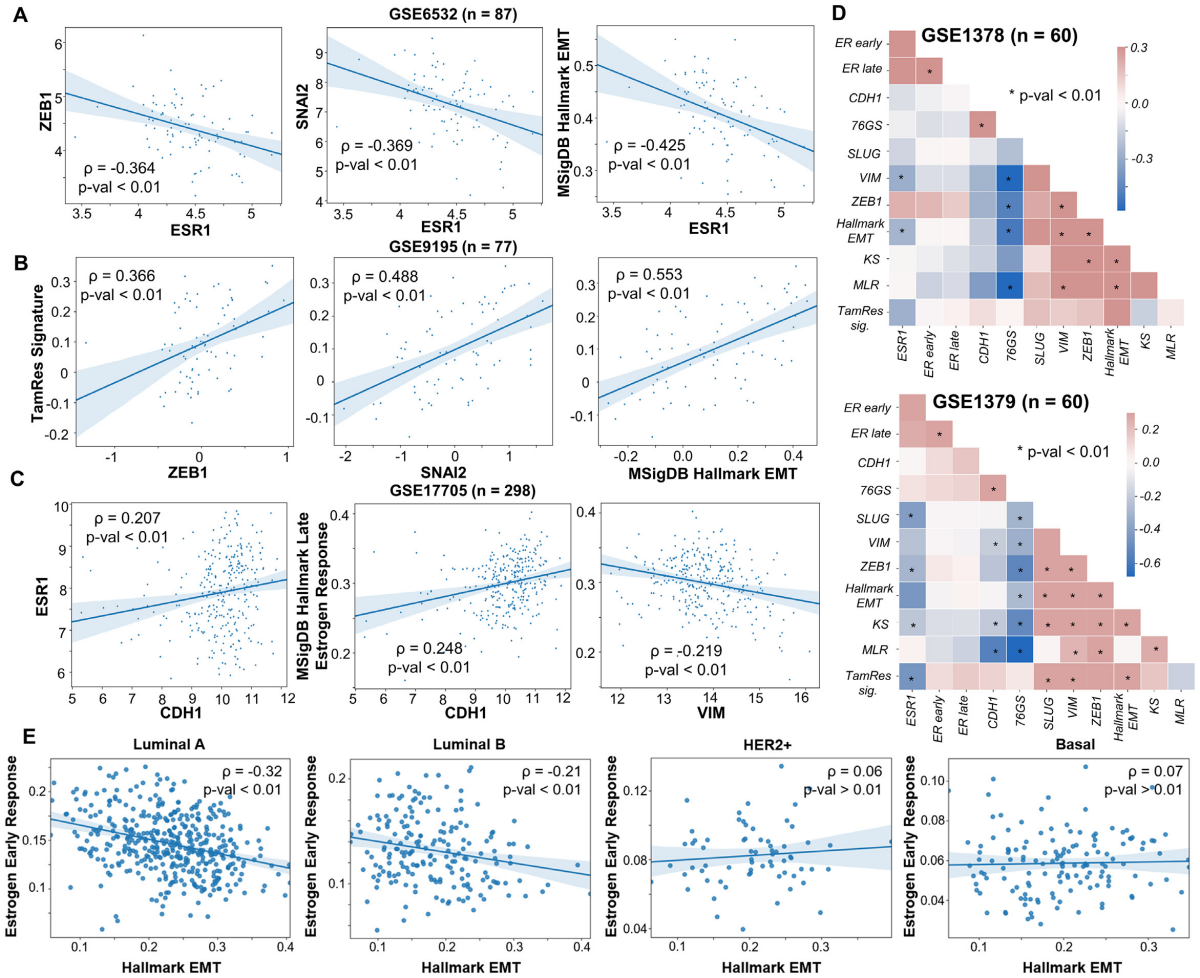
Gene expression analysis of publicly available datasets. (**A**) Correlation of ESR1 with ZEB1, SNAI2 (SLUG) and activity of MSigDB Hallmark EMT signature (ssGSEA scores) in a cohort of 87 ER+ breast cancer patients (GSE6532). (**B**) Correlation of tamoxifen resistance (ssGSEA scores) signature with expression of ZEB1 and SNAI2 and activity of hallmark EMT signature in primary breast tumors treated with tamoxifen in adjuvant setting (GSE9195). (**C**) Correlation of ESR1 expression levels and estrogen response activity with CDH1 and VIM in tumor samples from 298 ER+ patients treated with tamoxifen for 5 years. (**D**) Diagonal correlation matrix between expression levels (ZEB1, SLUG, VIM, CDH1, ESR1), EMT scoring metrics (76GS, MLR and KS) and gene set activity estimation (ER early response, ER late response, tamoxifen resistance, hallmark EMT signatures) for 60 samples of micro-dissected tumour biopsies (GSE1378) and whole tissue tumour biopsies (GSE1379) from a cohort of patients treated with tamoxifen for 5 years. (**E**) Correlation plots of estimated activities of estrogen response with hallmark EMT signatures in different subtypes of breast cancer in TCGA. Spearman correlation coefficient (ρ) and corresponding *P*-value are reported.

As a preliminary validation of our model predictions, we probed the association between EMT program and the loss in ERα activity with a concurrent gain in tamoxifen resistance markers. Specifically, we investigated clinical datasets of ER+ breast cancer patients treated with tamoxifen and observed that ESR1 (gene for ERα) levels were significantly negatively correlated with ZEB1 and SNAI2 (SLUG) levels, as well as with single-sample GSEA (ssGSEA) scores for MSigDB hallmark EMT signature (GSE6532; Figure 2A). Similarly, ssGSEA scores for signatures of resistance to tamoxifen at a proteomic level (TamRes) (36) were found to positively correlate with the levels of ZEB1, SNAI2 and MSigDB hallmark EMT program (GSE9195; Figure 2B). Further, CDH1, a well-known epithelial marker (E-cadherin), was found to positively correlate with ESR1 levels, as well as with ssGSEA scores for MSigDB late estrogen response. Consistently, VIM, a canonical mesenchymal marker, showed negative correlation with the ssGSEA scores for MSigDB late estrogen response (GSE17705; Figure 2C).

Next, for a more comprehensive analysis of such correlations, we investigated pairwise correlations among expression levels of ESR1, canonical mesenchymal (SLUG, VIM, ZEB1) and epithelial (CDH1) genes, three EMT scoring metrics (76GS, KS, MLR) (37) and four gene set activity estimation via ssGSEA scores (ERα early response, ERα late response, tamoxifen resistance and hallmark EMT). Across patient samples irrespective of whether the samples were micro-dissected tumour biopsies or whole tissue tumour biopsies, we observed an expected positive correlation among EMT metrics, mesenchymal markers and ssGSEA scores for hallmark EMT (GSE1378; Figure 2D). ESR1 gene expression levels usually correlated negatively with mesenchymal markers and/or EMT scoring metrics. Conversely, tamoxifen resistance signature correlated positively with the mesenchymal markers and EMT ssGSEA scores but negatively with ESR1 (Figure 2D).

Furthermore, we examined the subtype-specific trends in TCGA breast cancer data and observed that estrogen response and EMT exhibited robust negative correlation primarily in the two ER-positive subtypes (luminal A and luminal B) (51) but not in ER-negative subtypes (HER2+ and basal-like) (Figure 2E). Classification of luminal subtypes as ER-positive and that of HER2+ and basal-like as ER-negative is endorsed by recent transcriptomic profiling of 35 276 cells from 32 breast cancer cell lines capturing the percentage of cells positive for expression of ESR1 and various EMT players in cell lines representing different breast cancer subtypes. While luminal cell lines such as MCF7, BT474 and T47D have higher percentage of cells that are positive for ESR1 and the ones negative for SLUG and ZEB1, HER2+ and triple negative cell lines such as MDA-MB-453, BT549 and MDA-MB-468 showed a reverse trend (52). Put together, this analysis supports our prediction about an association between activation of EMT program and compromised ERα signaling activity in ER-positive breast cancer cases, supported both by bulk and single-cell data analysis.

### Reciprocal driving of the EMT program by suppressing estrogen receptor activity and *vice versa*

**Figure 3.**
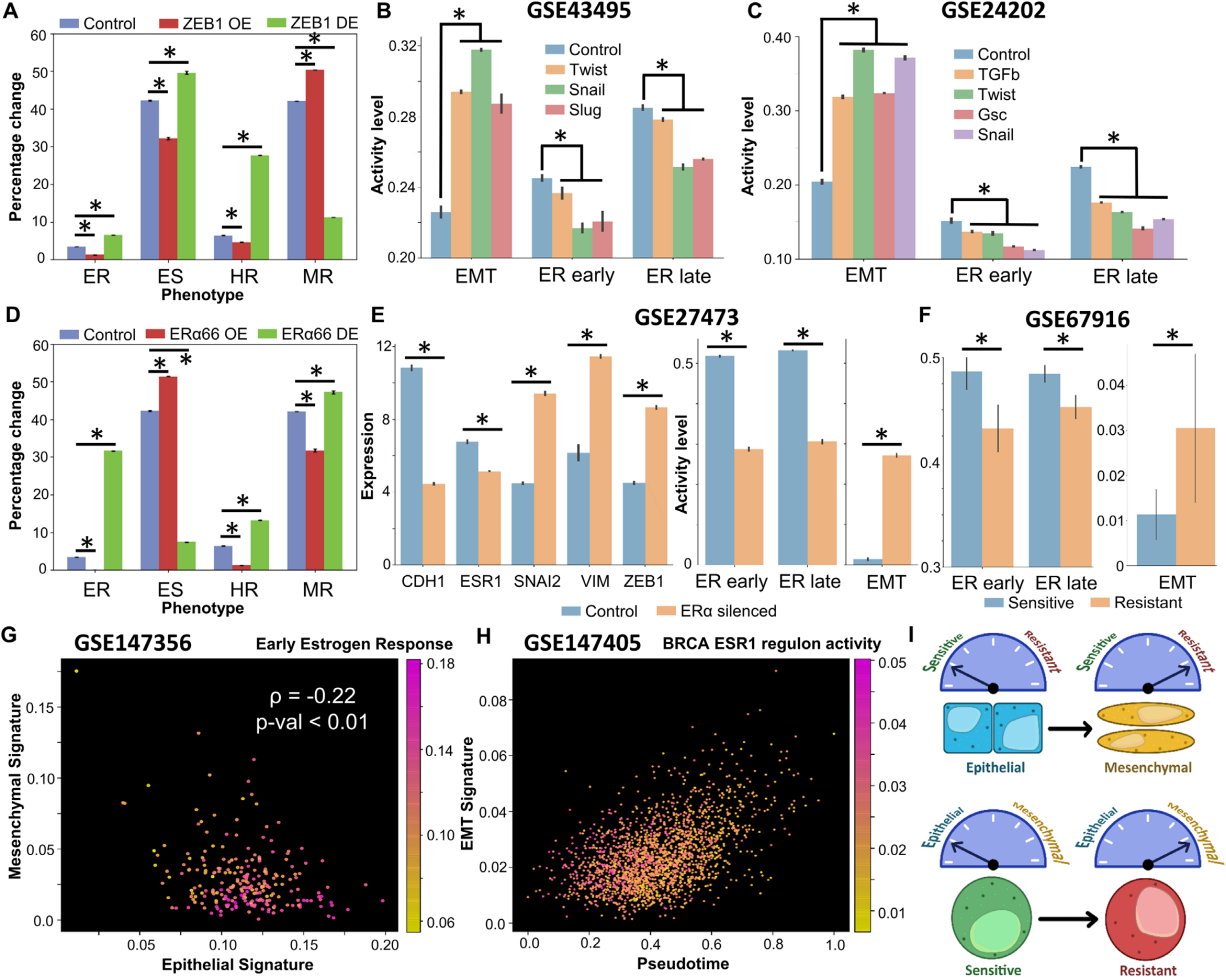
Induction of EMT can drive suppression of estrogen signaling and vice versa. (**A**) Impact of over-expression/down-expression of ZEB1 levels in RACIPE simulations on frequencies of different biological phenotypes. Error bars denote standard deviation across *n* = 3 replicates. (**B**) Experimental data (GSE43495) for EMT induction via Twist, Snail or Slug in HMLE cells and the concurrent decrease in the magnitude of early and late estrogen response. (**C**) Experimental data showing EMT induction via TGFβ, Twist, Gsc and Snail in HMLE epithelial cells and the concurrent decrease in the magnitude of early and late estrogen response (GSE24202). (**D**) Same as (A) but for over-expression/down-expression of ERα66. (**E**) Experimental data showing differences in gene expression levels of Cdh1, Vim, Snai2 (Slug), Vim and Zeb1 and change in magnitude of early and late estrogen response and the EMT program (ssGSEA on MSigDB hallmark EMT signature) in control and ERα silenced MCF7 cells (GSE27473). (**F**) Experimental data showing differences in activity levels of early ER response, late ER response and EMT program in sensitive and resistant MCF7 cell lines (GSE67916). For A–F, * denotes a statistically significant difference between the control and perturbed/induced case assessed by a two-tailed Student’s *t*-test assuming unequal variances. (**G**) Scatter plot showing association between activity of early estrogen response and cells with varying positions on a 2D epithelial–mesenchymal plane (GSE147356). Spearman’s correlation coefficient between epithelial and mesenchymal scores, and corresponding *P*-value are reported. Color bar represents the activity of early estrogen response. (**H**) Scatter plot showing activity of EMT signature in TGFβ treated MCF7 individual cells in pseudo time and the concurrent decrease in BRCA ESR1 regulon activity. Color bar represents the range of activity level of the ESR1 regulon (GSE147405). (**I**) Schematic showing bidirectional associations between the EMP and the drug resistance program, i.e. induction of EMT drives a switch to a therapy-resistant state, and acquisition of therapy resistance often drives EMT.

After investigating the correlations among EMT and tamoxifen resistance axes, we inspected whether these processes could drive one another. To understand the effects of perturbations of the EM axis on estrogen signaling axis and drug resistance, we first simulated over-expression (OE) and downexpression (DE) of ZEB1. ZEB1 OE led to a significant increase in the frequency of MR phenotype with concurrent decrease in ES, HR and ER phenotypes (Figure 3A). Opposite trends were seen in ZEB1 DE case, as expected. An increase in MR phenotype upon ZEB1 over-expression indicates that as cells are driven to undergo EMT via ZEB1, they lose their sensitivity to anti-estrogen drugs. This change should reflect as a decrease in estrogen signaling activity in cells. To test this hypothesis, we analyzed publicly available gene expression data for ER+ breast cells/cell lines induced to undergo EMT. In HMLE cells where EMT was induced by over-expression of Twist, Snail or Slug (53) (GSE43495); the ssGSEA scores for Hallmark EMT gene list showed significant increase in enrichment levels in the EMT program with a concurrent decrease in the activity of gene lists representing early estrogen receptor response and late estrogen receptor response (Figure 3B). Similarly, in other datasets, EMT induction via overexpression of TGFβ, Twist, Gsc or Snail or via downregulation of E-cadherin in HMLE cells (GSE24202) (54) showed reinforcing trends including enrichment of the tamoxifen resistance program (Figure 3C and Supplementary Figure S4A). Six1 over-expressing MCF7 (ER+ breast cancer cell line) cells (GSE23655) (55) showed similar trends (Supplementary Figure S4B). Consistently, over-expression of SNAIL in MCF10A (56) (GSE81929) upregulated ZEB1 and the EMT program and had downregulation of CDH1 levels as well as a concurrent drop in estrogen receptor activity (both early response and late response) (Supplementary Figure S4C). Interestingly, SLUG levels were reduced upon SNAIL over-expression, reminiscent of reports about mutual repression between SNAIL and SLUG (57). Given that SLUG can induce a partial EMT while SNAIL is likely to induce more of a complete EMT (48,58), these results together suggest that drug resistance can be achieved even through a partial EMT state, and that SNAIL and SLUG may follow different paths in the multi-dimensional EMT landscape, both of which can confer tamoxifen resistance to ER+ breast cancer cells.

Next, we inquired whether perturbing the levels of ERα66 could lead to a more mesenchymal phenotype by inducing either a partial or complete EMT. We simulated through RACIPE both the over-expression (OE) and down-expression (DE) of ERα66 and observed that ERα66 OE caused a marked reduction in levels of all resistant phenotypes with a concomitant increase in epithelial sensitive (ES) phenotypes (Figure 3D). Conversely, ERα66 DE caused a significant drop in the prevalence of ES phenotype with cells being pushed toward an ER, HR or MR phenotype (Figure 3D). Thus, the impact of ERα66 down-regulation can be multi-faceted where it could drive the system towards a more resistant state without changing the EMT status (i.e. to ER phenotype) or could push cells to a resistant state by making them more mesenchymal.

Interestingly, when ERα was silenced in MCF7 cells (24), there was a marked increase in the levels of mesenchymal genes such as SLUG, VIM and ZEB1 and a significant decrease in levels of CDH1. The effect of ERα silencing was also observed in decreased early and late estrogen receptor activities, and a concurrent increase in the activity levels of EMT hallmark gene signature (GSE27473; Figure 3E). This result establishes that ERα silencing alone can drive EMT and consequently exhibit a drug resistant phenotype. Further, in resistant MCF7 cells derived from sensitive parental population (59), the estrogen response pathways were found to be significantly suppressed, together with upregulated EMT program (GSE67916; Figure 3F). Proteomic analysis of MCF-7 cells resistant to tamoxifen were also found to exhibit enhanced migration, a hallmark of EMT (36). In another dataset consisting of sensitive and resistant cells (60), both CDH1 and ESR1 levels were lower in resistant cells and SLUG was significantly upregulated (GSE 26459; Supplementary Figure S4D). Although ZEB1 and VIM were not distinctly upregulated, the overall EMT program was higher in resistant cells than in sensitive breast cancer cells (Supplementary Figure S4D). Given the profiles of CDH1, ZEB1, ESR1 and VIM in these MCF7 cells, these cells could be construed as a hybrid-resistant (HR) phenotype, at least at a bulk level.

Further, we evaluated whether these trends were also preserved at a single-cell level. We first investigated the 10 cell sequencing data of ER+ breast cancer cells (61). The 2D scatter of activity levels of epithelial and mesenchymal genes revealed an expected negative correlation, denoting the reciprocal epithelial and mesenchymal phenotypes (GSE147356; Figure 3G). Intriguingly, we found that epithelial cells were more likely to harbor a responsive estrogen receptor pathway (Figure 3G). Further, the activities of early and late estrogen receptor hallmark genes showed a strong positive correlation with the 76GS EMT scoring method where higher scores indicate a more epithelial phenotype (Supplementary Figure S4E). The activity of BRCA specific ESR1 regulon was also found to be significantly positively correlated with 76GS EMT scoring metric, suggestive of an active estrogen receptor signaling in epithelial cells rather than mesenchymal ones (Supplementary Figure S4E). Finally, we examined whether EMT induction at a single cell level could itself drive a suppression of the estrogen receptor pathway activity. In MCF7 cells treated with TGFβ to induce EMT, we plotted the EMT program activity as a function of pseudo-time as reported earlier (62). EMT activity score showed a significant positive correlation with the pseudo-time, establishing pseudo-time as a proxy for EMT progression. Upon coloring by the BRCA-specific ESR1 regulon activity, we found that lower values of EMT activity were more likely to be associated with a higher ESR1 regulon activity level (GSE147405; Figure 3H). We confirmed this observation by plotting the activities of early and the late estrogen receptor hallmark genes and the ESR1 regulon with pseudo-time. All of them showed a significant negative correlation with pseudo-time, further supporting repression of these pathways alongside EMT (Supplementary Figure S4F).

Overall, we demonstrated that induction of EMT can suppress estrogen signaling axis and vice versa, resulting in concurrent change in cellular phenotypes along both the EMP axis and in levels of drug resistance in ER+ breast cancer cells (Figure 3I).

### Stochastic transitions between different phenotypes on EMT and drug resistance axes

Next, we investigated whether under the influence of biological noise (63), these different phenotypes can switch among one another on EMT and/or tamoxifen resistance axes. We identified the parameter sets simulated via RACIPE that gave rise to multi-stability (i.e. more than one steady state solutions, depending on the initial condition chosen) and performed stochastic simulations.

**Figure 4.**
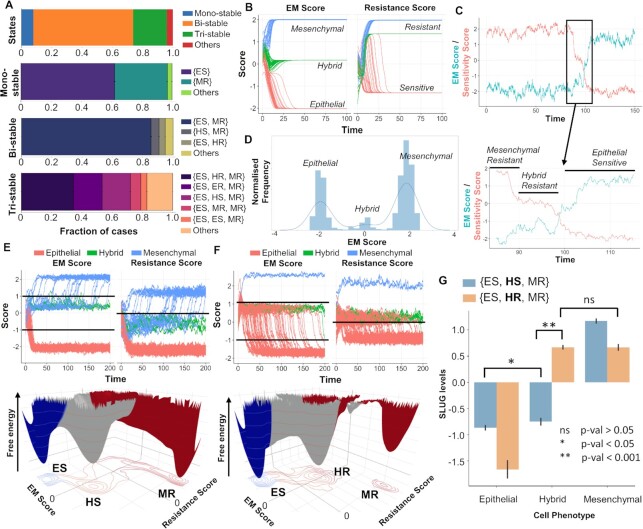
Stochastic stimulations showing dynamic state transitions among different biological phenotypes. (**A**) Fraction of RACIPE parameter sets resulting in monostable and multistable solutions (bi-, tri-, others) and the frequency distribution of phases that compose the monostable and multistable solution sets. (**B**) System dynamics for a representative {ES, HR, MR} parameter set showing the existence of the three biological EM phenotypes (E, H, M) and resistant (R) and sensitive (S) phenotypes when started from multiple initial conditions. (**C**) Time course showing the transition of the system from a MR to an ES phenotype through a HR state under the influence of noise. Sensitivity score is defined as negative of the tamoxifen resistance score, i.e. ERα66–ERα36. (**D**) Marginal distribution of the EM score from the time course shown in (C); three peaks denote existence of three distinct states along EM spectrum. (**E**) (top) Stochastic time series for multiple initial conditions tracking EM and Resistance scores in a representative parameter set from the {ES, HS, MR} phase. (Bottom) Landscape obtained by simulation of that parameter set with valleys representing stable states possible in the system. (**F**) Same as (E) but for a representative parameter set from the {ES, HR, MR} phase. (**G**) Changes in SLUG levels as the system transitions from ES to MR phenotype through either HS or HR state. HR state is characterized by high levels of SLUG compared to HS cell state. Student’s *t*-test results show the level of statistical significance between various comparisons.

We first characterized the proportion of parameter sets simulated by RACIPE that are monostable versus multistable. Approximately two-thirds (∼65%) of the parameter sets were found to be bistable, followed by 20% parameter sets exhibiting tristability, and 8% monostable cases (Figure 4A), indicating that the GRN simulated is poised for multistability. Within the monostable solutions obtained, {ES} and {MR} are the most predominant phases. Among bistable cases, the most common resultant phase is that of {ES, MR}, followed by {HS, MR} and by {ES, HR}. Finally, among tristable cases, {ES, HR, MR} phase is the most predominant followed by {ES, ER, MR} and then by {ES, HS, MR} (Figure 4A). Put together, these results suggest that epithelial sensitive and mesenchymal resistant are likely to be most frequent subpopulations in a given cancer cell populations, with smaller proportions of hybrid E/M cells which may be tamoxifen-sensitive or tamoxifen-resistant.

We focused on tristable parameter sets that contain ES and MR phenotypes and can enable transition between them through an intermediate state. We considered a representative parameter set from the {ES, HR, MR} phase and plotted the levels of EM score (ZEB1 – miR200 levels on log2 scale) and the resistance score (ERα36 – ERα66 levels on log2 scale), sampling multiple possible initial conditions. As expected, we observed three distinct levels in steady-state EM scores, corresponding to one each in the E, H or M region. The resistance score also showed three distinct levels; however, two of them were classified as R phenotype with one of them as S (Figure 4B). Stochastic simulations for this parameter set revealed noise-induced switching from a mesenchymal-resistant (MR) phenotype to an epithelial-sensitive (ES) phenotype through a hybrid-resistant (HR) phenotype (Figure 4C). The existence of these three states was further corroborated by plotting the marginal distribution of the EM Score obtained from the time profile that revealed three peaks with varying EMT scores (Figure 4D).

Next, we constructed landscapes to interpret cell-state transitions possible in the co-existing phenotypic combinations of {ES, HS, MR} and {ES, HR, MR}. To do so, we simulated the system from multiple initial conditions under the influence of noise and obtained the pseudo potential of the points in state-space as the negative logarithm of the probability of occurrence. We observed that for the representative case from the phase {ES, HS, MR}, trajectories that started out as hybrid-sensitive phenotype (HS) switched to a mesenchymal-resistant (MR) phenotype (Figure 4E, top), thus unraveling the dynamic nature of the observed cell states. The landscape constructed for this parameter set revealed three distinct valleys or ‘attractors’—ES, HS and MR (Figure 4E, bottom). For a representative parameter set from the phase {ES, HR, MR}, we again saw three distinct EM states and the corresponding expected resistant states (Figure 4F, top). The three ‘attractors’ observed here was shifted along the axis of the resistance score—from <0 for HS to >0 to HR (Figure 4F, bottom versus Figure 4E, bottom). Similar dynamics were observed for other kinetic parameter sets belonging to these two tristable phases (Supplementary Figure S5A, B). A key difference noted in the HS and HR states was the levels of SLUG, suggesting SLUG as a potential marker for hybrid E/M resistant phenotype (Figure 4G).

The analysis of these two tristable cases—{ES, HS, MR} and {ES, HR, MR}—shows that the phenomenon of acquiring a reversible resistance and a change in the EMT status, although correlated, can be semi-independent of one another i.e. first the cells can become hybrid E/M (but still sensitive) and then as they become mesenchymal, they acquire resistance traits or alternatively simultaneously first switch to a hybrid E/M resistant state and then an additional switch to a mesenchymal phenotype. However, cells can also switch directly between ES and HR states in bistable parameter scenarios (Supplementary Figure S5C–E).

These results demonstrate that the different states in the two-dimensional space of EMT and tamoxifen resistance (i.e. non-genetic heterogeneity) can also switch among one another (i.e. phenotypic plasticity) under stochastic variations in gene expression and/or biochemical rates. It should be noted that these state transitions can also be driven through external perturbations such as treatment with TGFβ or with anti-estrogen treatments such as tamoxifen. Simulations shown here demonstrate the paths cells traverse through while transitioning to other state(s).

### Complementary roles of heterogeneity and plasticity for the tumour survival of ER+ breast cancer cells under anti-estrogen treatments

After elucidating the emergent intra-cellular dynamics of the coupled gene regulatory network (GRN) of EMT and ERα signaling, we probed the effect of the dynamical traits of this network—phenotypic plasticity and nongenetic heterogeneity—at a cell population level. To gain a better understanding of how these two cell-autonomous traits, (i) heterogeneity in sensitivity of a cell toward a drug and (ii) ability of cells to switch bidirectionally between a sensitive and a resistant phenotype, can influence the long-term survival of a cell population, we developed a simple mathematical model involving only two components: drug sensitive (S) cells and drug resistant (R) cells. Previous modeling efforts in population dynamics have highlighted the importance of heterogeneity in enabling survival under various stressed conditions such as drug exposure, but they usually do not explicitly consider two subpopulations of cells (64–67).

**Figure 5.**
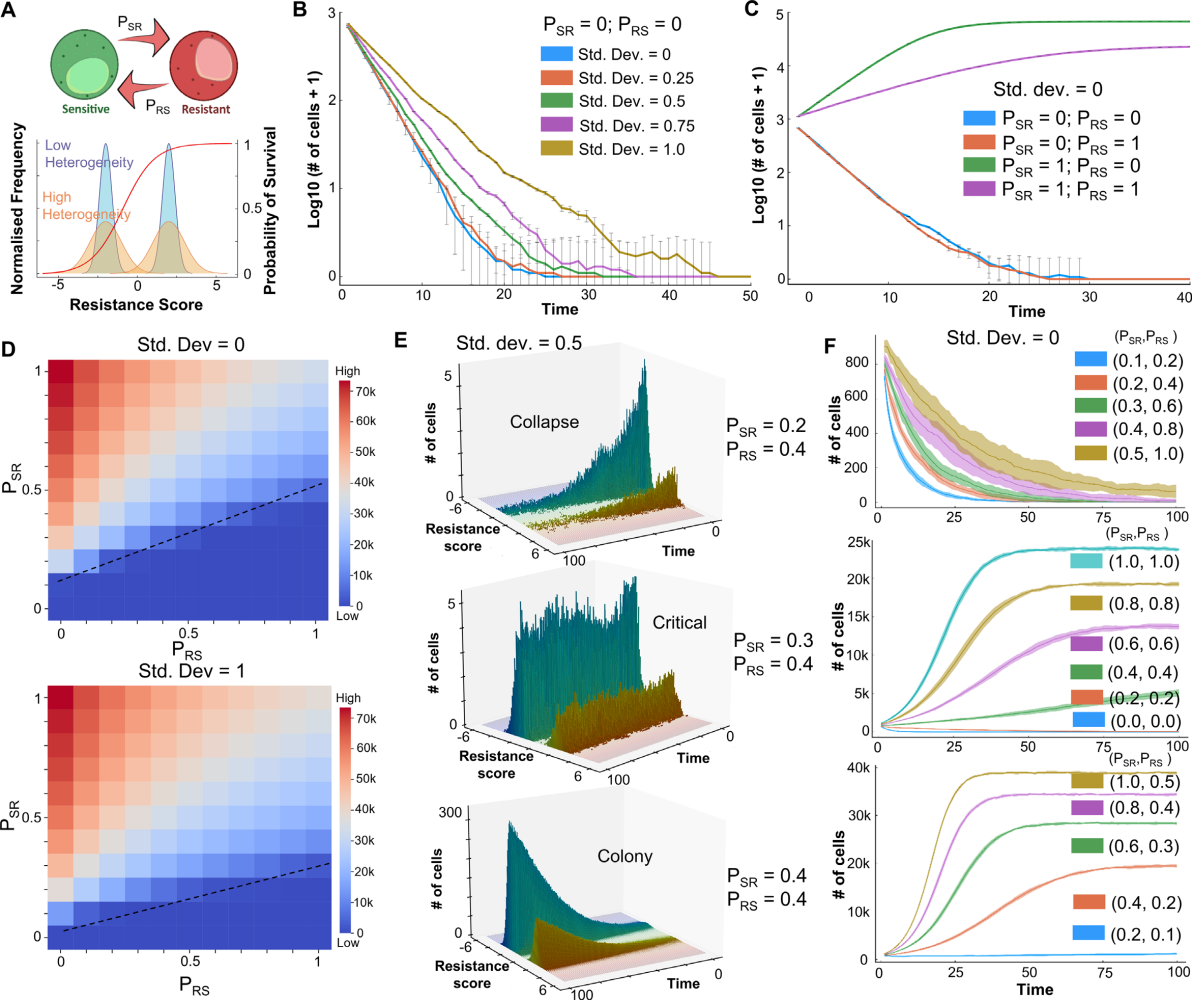
Effect of heterogeneity and plasticity on tumor survival in the presence of an anti-estrogen drug. (**A**) Schematic for model formulation showing inter-conversions between sensitive and resistant phenotypes (transition probabilities: *P*_SR_, *P*_RS_). Heterogeneity in the cell population is modelled by standard deviation (SD) of Gaussians from which resistance scores are sampled. Survival probability of cells is a function of resistance score approximated as a sigmoidal curve (shown in red). (**B**) Effect of heterogeneity on population sizes over time, starting with an initially all sensitive cell population and at *P*_SR_ = *P*_RS_ = 0. (**C**) Effect of plasticity on population sizes over time starting with an initially all sensitive cell population and no heterogeneity (SD = 0). (**D**) Population sizes (at time [*t*] = 100) as a function of *P*_SR_ and *P*_RS_ at two different heterogeneity levels, starting with an initially all sensitive cell population. Dotted lines indicate a qualitative boundary between tumor survival and elimination scenarios. (**E**) Distinct qualitative scenarios—collapse of initial population of cells, maintenance of the cell population around starting initial conditions (in the time frame considered) and net growth in a population of cells leading to survival of the tumor—at varying levels of *P*_SR_ and *P*_RS_. All simulations start with a fixed heterogeneity (SD = 0.5) and a fully sensitive population. (**F**) Population sizes over time as a function of varying values of (*P*_SR_, *P*_RS_). All simulations start with no heterogeneity (SD = 0) and a fully sensitive population. Shaded area represents the standard deviation around the mean of n=10 replicates.

In this modeling framework, cells can belong to either a sensitive (S) phenotype or a resistant (R) phenotype. The degree of sensitivity of a cell to a drug is defined by a ‘resistance score’ which is sampled from a Gaussian distribution with a given mean and standard deviation. Heterogeneity in the system is modelled via changing the standard deviation of the Gaussian from which the resistance score is sampled. The sensitivity of each cell (probability that the cell escapes drug-induced killing) depends on the resistance score through a sigmoidal curve (Figure 5A), reminiscent of typical IC50 curves. Further, the cells can switch from a sensitive to a resistant phenotype with a given probability *P*_SR_ and can switch back with a probability *P*_RS_, thus introducing plasticity into the system (Figure 5A). These probabilities can depend on various external conditions such as drug exposure time and/or drug concentration, but there is no noncell autonomous behavior such as cooperation and/or competition among the resistant and sensitive cell subpopulations (68–70).

Simulations for this model showed that in the absence of any drug, an initially fully sensitive population of cells with no heterogeneity (resistance score for all cells < 0, SD = 0), and no plasticity (*P*_SR_ = *P*_RS_ = 0) could grow to and eventually saturate to a population size close to the carrying capacity (10^5^ cells) (Supplementary Figure S6A; blue curve). However, the presence of drug can eliminate this population of non-plastic and homogeneous drug-sensitive cells (Supplementary Figure S6A; green curve); the rate of elimination depends on intrinsic growth and death rates of cells (Supplementary Figure S6A; orange and violet curves). Next, we characterized the role of increasing heterogeneity in a system devoid of plasticity (*P*_SR_ = *P*_RS_ = 0) i.e. for unimodal distributions. We observed that increasing heterogeneity (shown by different standard deviation values) can delay the time taken to eliminate a population of cells, but it was not sufficient in enabling the survival of population, as long as the population overall is predominantly sensitive as per the survival probability curve (Figure 5B; Supplementary Figure S6B–E). Furthermore, as reported earlier (64,71), for unimodal cell populations that are resistant, an increase in the variance can decrease the overall fitness of the population, contrary to what is observed for the sensitive populations (Supplementary Figure S6D).

Next, we examined the influence of plasticity (i.e. bimodal populations) in the absence of heterogeneity (SD = 0). For *P*_SR_ = 0 (no switching from a sensitive to a resistant phenotype), irrespective of the value of *P*_RS_, the cell population is eliminated (Figure 5C; blue and orange curves). For *P*_RS_ = 0 (no switching from resistant to sensitive population), if the probability of switching from sensitive to resistant is very high (*P*_SR_ = 1), the population of cells survive. However, increasing values of *P*_RS_ can decrease the final population size (Figure 5C; green and purple profiles). Further, we performed an exhaustive analysis of the *P*_SR_–*P*_RS_ plane with values ranging from 0 to 1 with steps of 0.1 and colored the matrix based on the final population size at time *t* = 100 steps. We first performed this analysis for two extreme values of heterogeneity (SD = 0 and 1). We observed that in presence of higher heterogeneity, population survival and growth was seen for more combinations of (*P*_SR_, *P*_RS_) values (compare Figure 5D; top and bottom panels). Further, if *P*_SR_ << *P*_RS_, an increase in heterogeneity alone cannot rescue the population and the population is eliminated. On the other hand, if *P*_SR_ >> *P*_RS_, the population survives and reaches near maximum colony sizes (close to carrying capacity in the system) (Figure 5D). These trends were qualitatively similar for other levels of heterogeneity (Supplementary Figure S7) as well, highlighting potentially universal organizing principles in determining cancer cell population fitness.

Finally, we characterized different qualitative properties exhibited by a cell population under varying values of *P*_SR_ and *P*_RS_ but at a fixed heterogeneity level. To visualize the proportion of cells in the sensitive and the resistant component separately, we plotted a 3D histogram showing the temporal evolution of cell population, colored by corresponding resistance scores. For an intermediate value of heterogeneity, depending on the relative values of *P*_SR_ and *P*_RS_, three distinct qualitative outcomes for cell population are possible: (i) it can be eliminated completely; (ii) it can maintain its critical population size similar in magnitude to the initial number of cells; and (c) it can increase rapidly to saturate at a higher value to establish a colony (Figure 5E). Next, we explored the effect of relative and absolute values of *P*_SR_ and *P*_RS_ on the overall population dynamics of the system. In absence of heterogeneity, as a representative case, we simulated various scenarios where the ratio *P*_SR_/*P*_RS_ was fixed to be 0.5, 1 or 2. When *P*_SR_/*P*_RS_ = 0.5, the population always collapsed irrespective of absolute values of *P*_SR_ and *P*_RS_ (Figure 5F). However, simulation cases that had higher absolute values of *P*_SR_ and *P*_RS_ showed a slower rate of extinction; this trend was seen also for *P*_SR_/*P*_RS_ = 1 and 2 (Figure 5F). These cases highlight that not only the relative rates of plasticity in either direction (S to R or vice versa) but also the absolute residence times (proxied by *P*_SR_ and *P*_RS_) can influence the population dynamics, by modulating the time of exposure of cells to the therapy. As expected, higher *P*_SR_/*P*_RS_ increases the propensity of population survival, and thus a faster growth curve and higher colony size. Collectively, these results indicate the complementary roles of heterogeneity and plasticity in the cell population to determine the survival probability of a population of cancer cells in the presence of an anti-estrogen therapy such as tamoxifen.

### ‘What does not kill [cancer cells] makes them stronger’—the influence of drug-induced plasticity and intrinsic non-genetic heterogeneity in population survival

**Figure 6.**
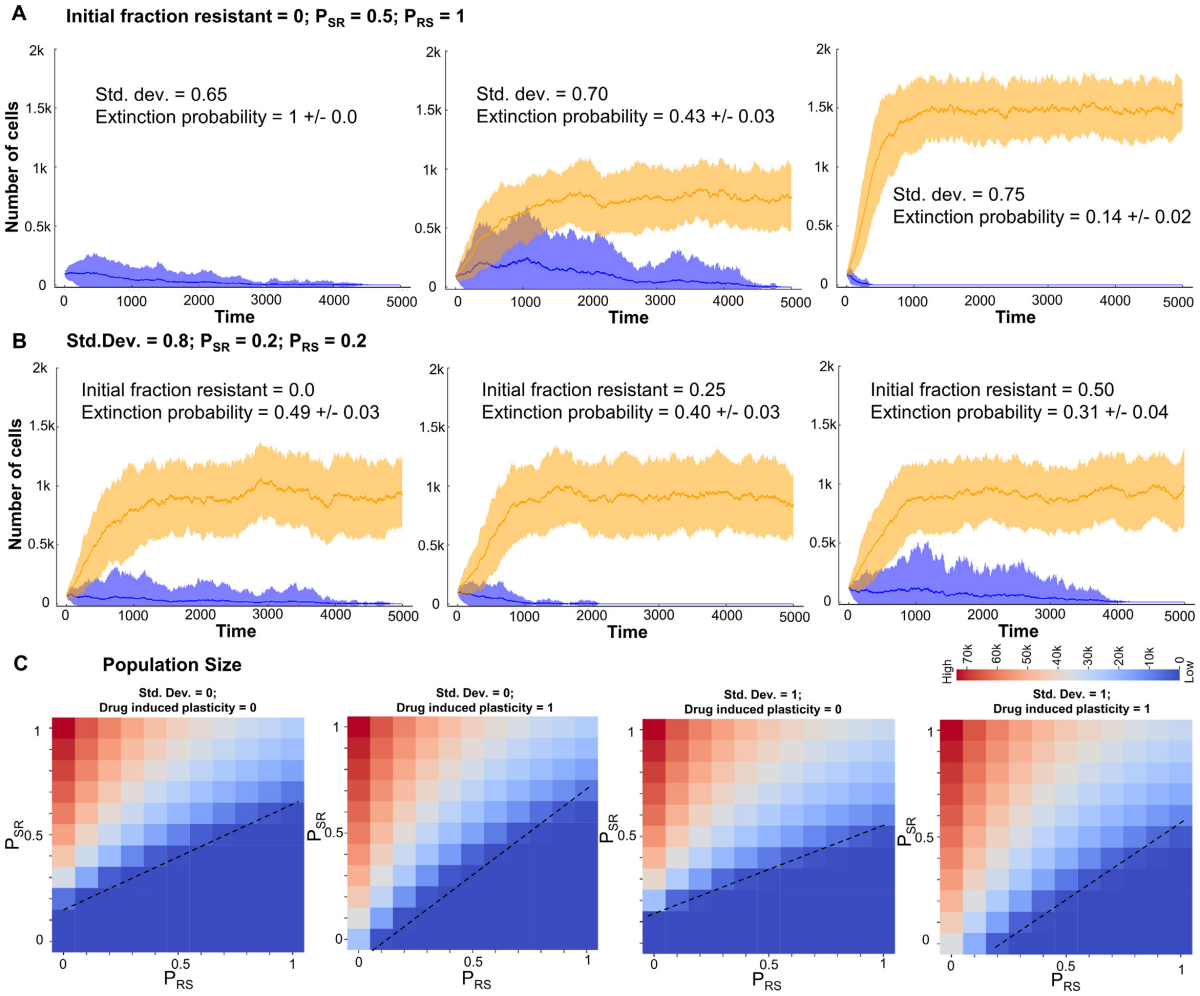
Different mechanisms promoting survival of a cancer cell population in the presence of an anti-estrogen drug. (**A**) Simulations showing increase in survival probability (decrease in extinction probability) of a cancer cell population with varying heterogeneity levels (SD = 0.65, 0.70 and 0.75) starting form an all sensitive population and fixed values of *P*_SR_ and *P*_RS_ at 0.5 and 1.0, respectively. (**B**) Simulations showing a modest increase in the survival probability of a cell population with varying levels of initially resistant cells (initial fraction = 0.0, 0.25 and 0.50) and fixed values of *P*_SR_ and *P*_RS_ at 0.5 and 1.0, respectively. Orange ribbon represents the collection of all states that survive and form a colony and blue ribbon for all those cases that are eventually eliminated. The dark line represents the mean and the band represents the SD around that mean for an ensemble of simulations. (**C**) Final population sizes (at time [t] = 100) as a function of *P*_SR_ and *P*_RS_ at two different heterogeneity levels (SD = 0, 1) and two different levels of drug induced plasticity (0 and 1) starting with an initially all sensitive cell population. Dotted lines indicate a qualitative boundary between cases where a tumour survives or is eliminated by the presence of the drug.

As described above, depending on the absolute values of *P*_SR_ and *P*_RS_, a population of cells can collapse, grow a colony or maintain the population size around the initial value. The last case is likely to be a metastable state as under the effect of intrinsic noise, the population can be pushed to be eliminated completely or grow and saturate to carrying capacity of the system. This feature enables us to define the extinction probability for a cell population for a set of simulations from specified initial conditions. Extinction probability is defined as the fraction of cases in which the population is eliminated after a long time. For most values of *P*_SR_ and *P*_RS_, the extinction probability is either 0 (population always grows and establishes a fixed colony) or is 1 (population is eliminated), under the influence of no heterogeneity (SD = 0). We explored whether increasing heterogeneity can have an extinction probability between 0 and 1. As a representative case, we found that starting from an initially all sensitive population (resistance score < 0), with *P*_SR_ = 0.5 and *P*_RS_ = 1, intermediate heterogeneity level (SD = 0.65) led to extinction of the population (Figure 6A, left; extinction probability = 1.0). However, increasing the heterogeneity (SD = 0.7) led to a decrease in extinction probability (0.43 ± 0.03) with the surviving cases saturating at a population level of ∼750 cells (Figure 6A, middle). A further increase in heterogeneity further reduced extinction probability (0.14 ± 0.02) concomitant with an increasing saturating population size of ∼1500 cells (Figure 6A, right). These simulations illustrate a protective role of non-genetic heterogeneity in maintaining a cancer cell population, under the influence of therapy, especially at high enough intrinsic switching rate of cells to a resistant state, due to stochastic dynamics of phenomenon such as EMT.

Next, we investigated the effect of initial fraction of ‘pre-existing resistant cells’ (72) on the population extinction probability. At fixed heterogeneity (SD = 0.8) and plasticity levels (*P*_SR_ = *P*_RS_ = 0.2), starting with only sensitive cells (resistance score < 0), the extinction probability was 0.49 ± 0.03 but increasing the initial fraction to 0.25 (i.e. 25% of initial cells have resistance score > 0) caused a modest decrease in extinction probability (0.40 ± 0.03) (Figure 6B), as compared to the impact of heterogeneity, where a decrease of approximately 60% was noted for extinction probability (Figure 6A). A further increase in this initial fraction also had a similar weak effect in changing either the extinction probability (0.31 ± 0.04) or the final population size (Figure 6B). This analysis revealed that the initial fraction of reversibly resistant cells is perhaps a weaker factor, especially at lower intrinsic transition probabilities between sensitive and resistant cells.

Finally, we investigated the effect of drug-induced plasticity (i.e. externally induced transitions from a sensitive to a resistant state) (9) on the cell population dynamics. In our model, sensitive cells that can survive drug exposure in stipulated time have a probability to switch to resistant state. We monitored the final population size with varying levels of drug induced plasticity (0 and 1) at two different levels of heterogeneity (SD = 0 or 1). We observed that under high drug-induced plasticity conditions, especially with smaller *P*_RS_ values, a marked increase in final population size was observed, irrespective of the extent of heterogeneity (Figure 6C and Supplementary Figure S8).

Put together, these observations show diverse mechanisms that can promote the survival of a cancer cell population under the influence of various anti-estrogen drugs: (i) nongenetic heterogeneity in initial cell population, including the percentage of *de novo* reversibly resistant cells and (ii) stochastic switching between cell states (ES, MR) under the influence of noise, maintaining a dynamic equilibrium of sensitive and resistant cells and (ii) drug-induced switch to a reversible resistant state for cells that do not die upon exposure of the drug, i.e. cells that tend to follow the Nietzsche's proposal of ‘what does not kill me makes me stronger’ (73).

### Combinatorial strategies to target a plastic and heterogeneous cancer cell population

After deciphering multiple possible routes to long-term sustenance of a resistant population which can lead to tumor growth, we investigated what mechanisms can be efficiently deployed to target a plastic and heterogeneous cancer cell population. From our network simulations and follow-up analysis of bulk and single-cell transcriptomic data, we established a consistent association between epithelial state and higher drug sensitivity, as well as a mesenchymal one exhibiting recalcitrance to tamoxifen (Figures 1 and 2). Thus, in our cell population dynamics framework, we incorporated the effect of an MET-inducer, i.e. an external signal that increased *P*_RS,_ just as drug-induced switching increased *P*_SR_. Thus, this MET-induced sensitivity forced cells to switch from a more mesenchymal (i.e. resistant) to an epithelial (i.e. sensitive) cells, thus reducing the fraction of resistant cells in the population.

**Figure 7.**
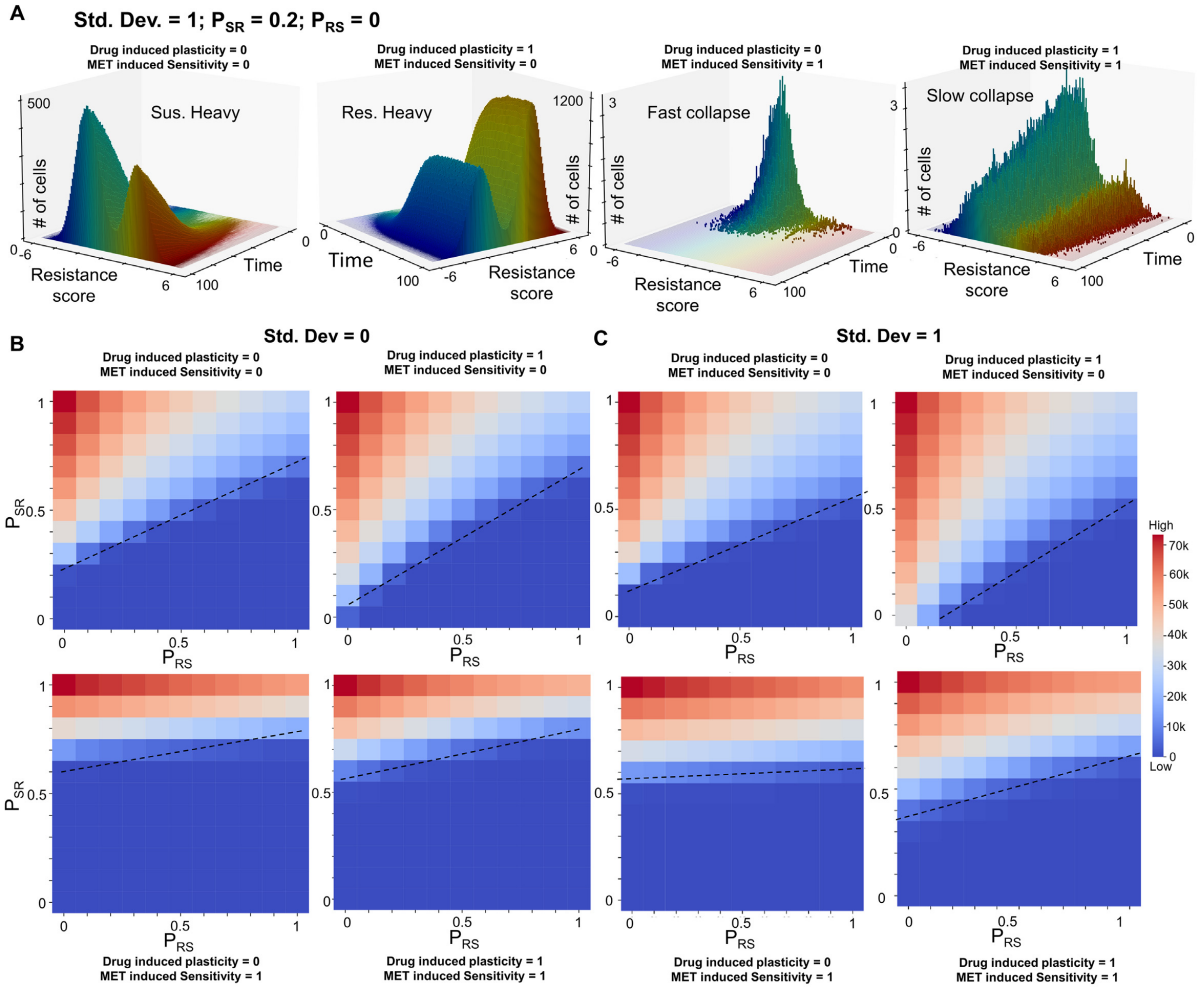
MET inducer, in conjunction with anti-estrogen drugs, can potentially limit the survival of cancer cell population. (**A**) Temporal evolution of a population of cancer cells (resistant and sensitive both) under different levels of drug induced plasticity (0 and 1) and MET induced sensitivity (0 and 1). Simulations were performed starting form an all sensitive population and fixed values of *P*_SR_ and *P*_RS_ at 0.2 and 0, respectively. (**B**) Final population sizes (at time [t] = 100) as a function of *P*_SR_ and *P*_RS_ two different levels of drug induced plasticity (0.1 and 1) and two different levels of MET induced drug sensitivity (0 and 1) starting with an initially all sensitive cell population with no heterogeneity in the system. Dotted lines indicate a qualitative boundary between cases where a tumour survives or is eliminated by the presence of the drug.

To simulate the effects of such a scenario, we first chose a representative parameter set that enabled a surviving population of cells, with a larger frequency of sensitive cells than resistant ones (Figure 7A, i). Upon introducing drug-induced plasticity into the system, we observed faster saturation, and a changed demographic with most of the population consisting of resistant cells (Figure 7A, ii). Upon introduction of the MET inducer in absence of drug-induced plasticity, the population undergoes a rapid collapse as the cells are sensitized to the drug, causing drugs to kill the cells (Figure 7A, iii). However, in the presence of drug induced plasticity, the population undergoes a relatively slower collapse as these two factors (drug-induced plasticity and MET-induced sensitivity) are opposing in nature (Figure 7A, iv).

This behavior is generally conserved across multiple parameter values corresponding to heterogeneity and plasticity (SD, *P*_SR_, *P*_RS_). Irrespective of the extent of heterogeneity, we see a larger combination of (*P*_SR_, *P*_RS_) values in the *P*_SR_–*P*_RS_ plane allowing for population collapse in the presence of MET-induced sensitivity as compared to the control case (Figure 7B). These observations suggest that strategies that can revert the influence of therapy-induced plasticity to alter the population demographic to a higher proportion of sensitive cells can be applied together with canonical anti-estrogen therapies to halt the dynamic adaptability of cell population, thus ‘trapping’ them in a drug-sensitive state. In the current paradigm, ‘tolerant’ cells that are able to escape being killed upon drug exposure are very likely to switch to a resistant state, given the coupled EMT-estrogen signaling coupling, thus adding to existing tumor burden. However, alleviating this side-effect of the drug-induced plasticity through MET inducers that may sensitize the population can be a more efficient therapeutic combination.

## DISCUSSION

Drug resistance, similar to many hallmarks of cancer, has traditionally been presumed to have a strong genetic underpinning. Thus, drug-resistant cells in a tumor population have been thought of containing pre-existing (*de novo*) mutations or in acquired mutations during the course of therapy (2). However, over the past decade, accumulating experimental/preclinical and clinical evidence of nongenetic, stochastic and reversible modes of drug resistance—labeled often as drug-tolerant persisters (DTPs)—have been reported *in vitro* and *in vivo* (5,74–77), reminiscent of similar observations in microbial systems (78). DTPs have been also shown to give rise to stable drug-resistant phenotypes involving genetic changes upon prolonged growth (77), highlighting the importance of realizing different timescales over which cellular adaptation can occur. While short-term changes are often phenotypic in nature and involve foraging of the phenotypic landscape, long-term survival strategies may involve genomic changes to enable being trapped in the ‘attractors’ explored during short-term foraging. While such transitions to a persister (reversibly resistant) cell state have been largely associated with epigenetic reprogramming (2), recent studies show significant variation in transcriptional program of such cells too as compared to sensitive cells from the same genetic background (72,79–81), suggesting a critical role of transcriptional regulation as well in the emergence of reversible therapy-resistance (i.e. persistence), a ‘bet-hedging’ strategy to ensure survival in time- and/or space-varying environments (78).

**Figure 8.**
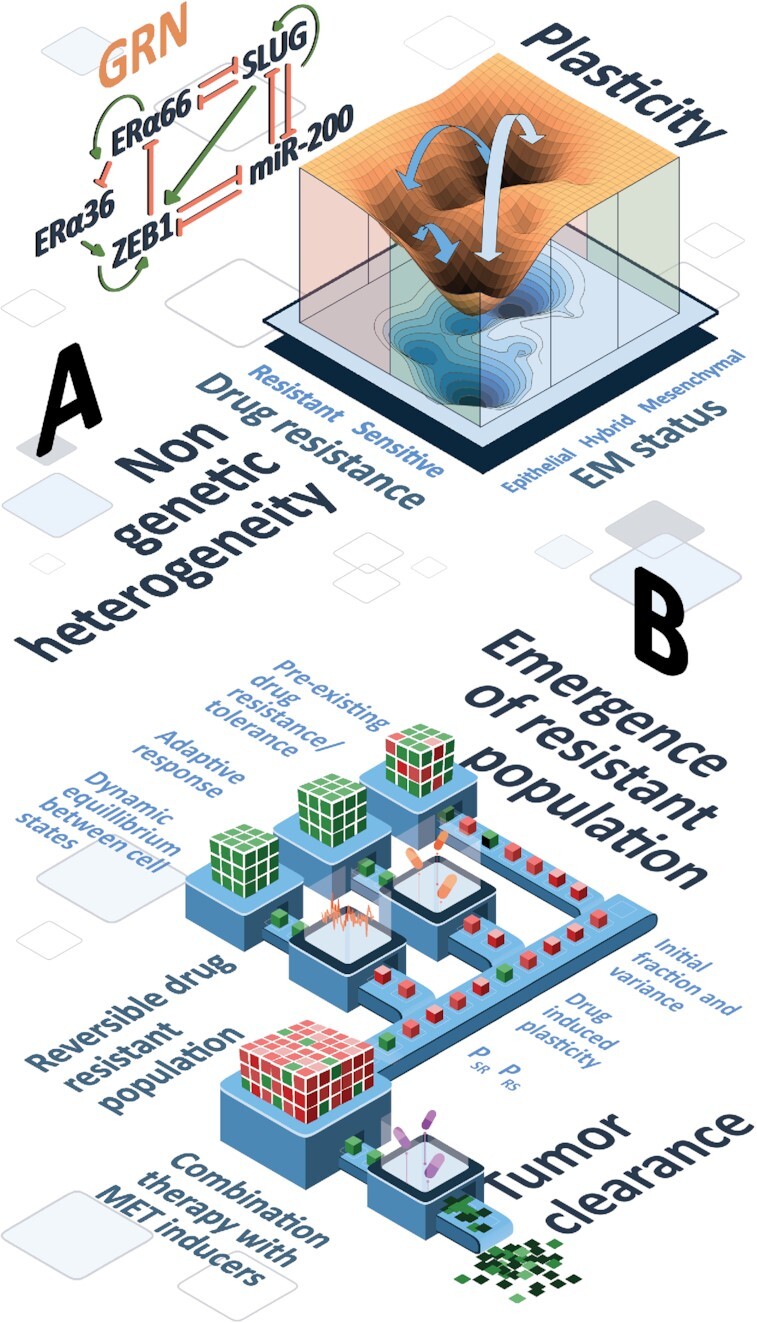
Schematic depicting dynamical traits of coupled EMT-ERα signaling network and its implications in tumor survival. (**A**) Landscape showing multiple phenotypes defined on EMT and drug resistance axes: ES (epithelial-sensitive) and MR (mesenchymal-resistant) phenotypes are more dominant (witnessed by depth of the valley in the landscape). Arrows induced transitions under the influence of noise or drug among six phenotypes (ES, ER, HS, HR, MS, MR). (**B**) Population dynamics showing multiple parallel paths to long-term resistance (pre-existing reversibly resistant cells, stochastic switching and dynamic equilibrium, and drug-induced plasticity) and the predicted effect of combinatorial therapy (anti-estrogen therapy + MET inducers) to drive population collapse.

Here, we have identified one such transcriptional program in ER+ breast cancer cells that can cause a reversible resistance to the treatment of tamoxifen, an anti-estrogen therapeutic. Our results suggest that EMT and resistance to tamoxifen can drive each other; thus offering a mechanistic explanation for empirical observations showing that cells undergoing EMT are more resistant to tamoxifen (20) and that tamoxifen-resistant cells are EMT-like (21,25). Notwithstanding this broad association between the two axes, which was validated in bulk and single-cell transcriptomic data, our model predicts the co-existence of and stochastic switching among six possible phenotypes—epithelial sensitive (ES), epithelial resistant (ER), hybrid sensitive (HS), hybrid resistant (HR), mesenchymal sensitive (MS) and mesenchymal resistant (MR) (Figure 8A). Thus, a progression to a full EMT need not be required for acquiring resistance to tamoxifen, and the association between at least a full EMT and drug resistance can be semi-independent, as seen for coupled EMT-stemness dynamics *in vitro* and *in vivo* (82,83). Our stochastic simulations showed that cells can switch from an ES to MR state via HS or HR states, highlighting two alternative paths to acquire resistance. But whether cells show hysteresis in these paths (i.e. how reversible or irreversible can such transitions be) need to be investigated more carefully experimentally. Future efforts should focus on drawing more comprehensive landscapes for transitions along coupled axes of plasticity such as EMT, immune evasion, stemness and metabolic reprogramming (84), besides drug resistance. We also proposed SLUG as a marker for hybrid E/M and tamoxifen resistant phenotype, which may help explain association of worse clinicopathological features with high SLUG levels (48).

While our model extensively features the connections among five players (ZEB1, SLUG, miR-200, ERα66 and ERα36) transcriptionally and their corresponding emergent phenotypes and dynamics, development of EMT and/or drug resistance is a much more complex process. Furthermore, the mechanism of drug resistance would largely depend on the mechanism of action of the drug. For example, aromatase inhibitors, a major first line endocrine therapy for postmenopausal metastatic ER+ breast cancer that operate via inhibiting estradiol synthesis rather than by inhibiting ERα66 (85,86) may not follow the mechanisms of reversible drug resistance as proposed here. The scope of our model is limited to drugs directly affecting the abundance or functionality of the ERα66 itself (e.g. fulvestrant and tamoxifen). Moreover, although our model does not explicitly consider it, we are not excluding the possibility of acquired genetic mutations in ESR1 or other genes which may alter the network dynamics. Similar to observations in lung cancer (87), the role of genetic mutations in contributing to acquired resistance in ER-positive breast cancer also becomes apparent only at longer time-scales ranging over months (88), instead of instant adaptation that may be offered by transcriptional and/or epigenetic reprogramming as represented through emergent dynamics of the network investigated here.

Despite these limitations, the model simulations offer profound conceptual contributions. First, we postulate a putative mechanism that can explain diverse experimental observations and proposes a mutual dependence of two axes of plasticity that are crucial in cancer progression. Such models can help guide future experimental studies to investigate this coupling more comprehensively. Second, these specific molecular players can be thought of representative of various functional modules, and the fundamental feature of multistability demonstrated here can help conceptualize reversible phenotypic plasticity and non-genetic heterogeneity along multiple axes, as live-cell imaging and single-cell RNA-seq data becomes more prominent. Third, the population dynamics framework used here offers insights into how bidirectional plasticity and non-genetic heterogeneity can contribute to survival of a tumor cell population as well as the phenotypic demographic of the tumor colony.

The population dynamics framework has been maintained intentionally simple. Such simple frameworks are quite powerful in explaining many experimental phenomenon, as shown recently by Rehman *et al.* (75), where they could explain the maintenance of clonal complexity after drug treatment in tumors *in vivo* using a population dynamics model that assumes that all cells in the population are equally potent to switch to a reversibly resistant state and the choice of cell survival is completely independent of genetic background. One complexity that can be added to our model is the effect of ecological interactions between the two species—sensitive and resistant cells—such as cooperation or competition for resources (68–70). Other adaptation of the model can be to include cell-state transition probabilities as a function of relative stability of states calculated from the landscape estimated for an intracellular GRN. Similarly, death rate incorporated in our model can depend on internal challenge faced by cells during the metastatic cascade, such as anoikis and/or interaction with various immune cells.

Our population dynamics framework suggests that phenotypic plasticity (reversible transitions between drug-resistant and -sensitive phenotypes) and non-genetic heterogeneity (pre-existing reversibly resistant cells) can promote the survival of tumor population under drug treatment, with a higher contribution coming from plasticity, especially a switch from sensitive to resistant state. While heterogeneity aids in tumor survival, plasticity is crucial for the effects of heterogeneity to have a significant impact in terms of tumor fitness and survival. We found that the tumor size is maximum at an optimal plasticity level, beyond which the effect of plasticity, while still present, is reduced. These observations are consistent with reports about intermediate levels, not extremely high levels, of chromosomal instability being associated with the worst clinical outcomes (89). Furthermore, composition of final tumor population after a prolonged treatment was found to be variable from purely resistant to purely sensitive. Overall, the maintenance of long-term ‘resistance’ can be achieved via multiple paths: (i) non-genetic heterogeneity in initial cell population, (ii) stochastic transitions among sensitive and resistant states driven by processes such as EMT, maintaining a dynamic equilibrium of cell subpopulations (30) and (iii) drug-induced transitions to a reversibly resistant state (Figure 8B).

Based on these features, we conceptually integrated the effect of MET on drug resistance, i.e. the broad association of an epithelial phenotype with a drug-sensitive state. We observed that MET inducers can minimize the impact of switch from sensitive to resistant phenotype and can thus counteract the impact of both drug-induced plasticity and population heterogeneity to a large extent. Whether such combinatorial strategies are likely to be more effective simultaneously or sequentially (90,91) is a question beyond the scope of this study, but can be answered by developing a multi-scale model including non-cell-autonomous effects such as cooperation or competition, building on the principles of multistable regulatory networks and consequent phenotypic plasticity and non-genetic heterogeneity elucidated here. Future efforts to improve the predictability of such models will require rich dynamic experimental data based on which the observed cellular behavior can be realized in hyperspace of phenotypes obtained via mechanism-based and/or data-based investigation of regulatory networks.

## DATA AVAILABILITY

All codes for the manuscript are available at: https://github.com/csbBSSE/TamRes.

## Supplementary Material

zcab027_Supplemental_Files
